# A FOCUS-PDCA quality improvement model for reducing the distribution defect rate of sterile packages

**DOI:** 10.1038/s41598-023-42295-8

**Published:** 2023-09-12

**Authors:** Yongdeng Huang, Yan Huang, Liangying Yi, Wei Pan, Yanhua Chen

**Affiliations:** 1 Department of Sterile Processing Nursing, West China Second University Hospital, Sichuan University/West China School of Nursing, Sichuan University, Chengdu, Sichuan China; 2 Key Laboratory of Birth Defects and Related Diseases of Women and Children (Sichuan University), Ministry of Education, Chengdu, Sichuan China; 3 Department of Nursing, West China Second University Hospital, Sichuan University/West China School of Nursing, Sichuan University, Chengdu, Sichuan China

**Keywords:** Health care, Risk factors

## Abstract

This study aimed to investigate the effect of the application of the FOCUS-PDCA quality improvement model in terms of reducing the distribution defect rate of the sterile packages processed by the CSSD. The FOCUS-PDCA quality improvement model was applied to analyze the causes of the distribution defects of sterile packages, develop improvement measures, and compare the distribution defect rates before and after the application of the FOCUS-PDCA model. Following implementation of the FOCUS-PDCA quality improvement model, the distribution defect rate of sterile packages decreased from 1.74 to 0.37% (*P* < 0.05). The FOCUS-PDCA quality improvement model can produce a substantial reduction in the distribution defect rate of sterile packages, ensuring the quality of sterile supplies.

## Introduction

In the modern clinical setting, the process of distributing sterile packages to clinical departments must adhere with strict health and safety guidelines^1^. These guidelines concern quality inspection, scanning, transit, loading and transportation of sterile packages^2^. The national health standard (WS310.2-2016) for China^3^ stipulates that the validity and packaging integrity of sterile packages should be checked prior to distribution. The distribution of sterile packages is the last line for sterile item management in the central sterile supply department (CSSD). It is crucial for the prevention and control of nosocomial infections^4^. According to numerous studies in China and abroad^5, 6^, the distribution defect rate of sterile packages falls within a range of 0.75–0.98%. Our hospital’s CSSD staff conducted Pareto analysis on our CSSD work quality data for the period from 2018 to 2020. We found that the distribution defects of sterile packages accounted for 56% of CSSD work quality defects and the distribution defect rate was 1.74%, higher than that reported in China and other countries. Therefore, our CSSD launched a quality improvement study to reduce the distribution defect rate.

The FOCUS-PDCA quality improvement model was created by the Hospital Corporation of America^7, 8^. It is an extension of the PDCA cycle, aiming to analyze and explore the problems existing in the work procedure in a more detailed way, so as to improve the work quality. It contains nine steps, symbolized by the following: Find (F) a problem to improve; Organize (O); Clarify (C); Understand (U); Select (S); Plan (P); Do (D); Check (C); and, Act (A)—condensed in the acronym, FOCUS-PCDA. The FOCUS-PCDA quality improvement model has been widely applied in many fields.

Ding et al.^9^ used the bundle strategy based on FOCUS-PDCA to carry out nutritional intervention on patients with neurocritical illness. They found that it could significantly improve the body’s nutrition, reduce the risk of malnutrition and enteral nutrition-related complications, and improve the body’s immune function. Chen^10^ utilized FOCUS-PDCA to find out the reasons why the filing rate of medical records did not meet requirements, thereby improving work procedures.

Studies on the application of FOCUS-PDCA quality improvement model in CSSD are rare, and there were no studies on the application of this model in sterile package distribution. In this study, we aim to investigate the effects of the application of the FOCUS-PDCA quality improvement model in terms of reducing the distribution defect rate of the sterile packages processed by the CSSD in our hospital.

## Methods

A total of 16,480 sterile packages distributed during May and June 2021, prior to application of the FOCUS-PDCA quality improvement model, were classified as the control group. A total of 15,976 sterile packages distributed during October and November 2021, during application of the FOCUS-PDCA quality improvement model, were classified as the observation group. Data concerning the numbers of distributed sterile packages were exported from the CSSD’s information system. The CSSD quality controllers identified and recorded the distribution defects.

The inclusion criteria of sterile items were as follows: only items that were cleaned, packaged and sterilized by our hospital’s CSSD were considered. Exclusion criteria covered disposable sterile items; sterile items that were transported to our hospital for sterilization after being cleaned and packaged by CSSD of other hospitals; and, items handled by medical device companies.

### Criteria for distributing sterile packages

According to the Chinese national health standards^11^, sterile packages should meet the following conditions when distributed to the clinic departments: conforming packaging and sterilization, complete label-based information outside the package, accurate distribution to the specified clinical department, and accurate quantity of the items distributed. If the above conditions are not met, a distribution defect of sterile packages is said to have arisen.

### Application of FOCUS-PDCA quality improvement model

#### Find (F)

The CSSD distributes sterile packages to the clinical departments. When using the sterile items, the clinical departments check whether the packaging of sterile items has been damaged, whether the quality of sealing fails to conform with the criteria, and whether the label information outside the package is illegible. Of the 16,480 sterile packages distributed during May and June 2021, 287 packages with distribution defects were identified (defect rate of 1.74%). According to the survey of clinical departments’ satisfaction on the CSSD work during the same period, the average satisfaction score was (95.53 ± 0.24).

#### Organize (O)

The Continuous Quality Improvement (CQI) team in our hospital consists of 8 members, including the head nurse of the nursing department, head nurse of the CSSD, quality controllers, head of the distribution area, and head of the operating room nursing group. All of them are proficient in the national health standards of the CSSD and the distribution and management rules of sterile packages.

#### Clarify (C)

Following a discussion within the CQI team, we designed a form to collect data detailing the occurrence of different forms of distribution defects among the sterile packages. The distribution defects of sterile packages are classified into the following categories:

1. *Seal quality defects*: plastic punch is not sealed, sealing is incomplete, or sealing strip has wrinkles and bubbles;
2. *Defects of label outside the package*: package name on the label is inconsistent with the items inside the package, or label information is incorrect, illegible or incomplete;
3. *Packaging material defects:* packaging material is contaminated, damaged or incorrect;
4. *Sterilization quality defects:* sterilization physical monitoring, chemical monitoring and biological monitoring has failed; occurrence of wet packs;
5. *Distribution error:* package distributed to incorrect clinical department; quantity of items inside the package is incorrect.

**Figure 1:**
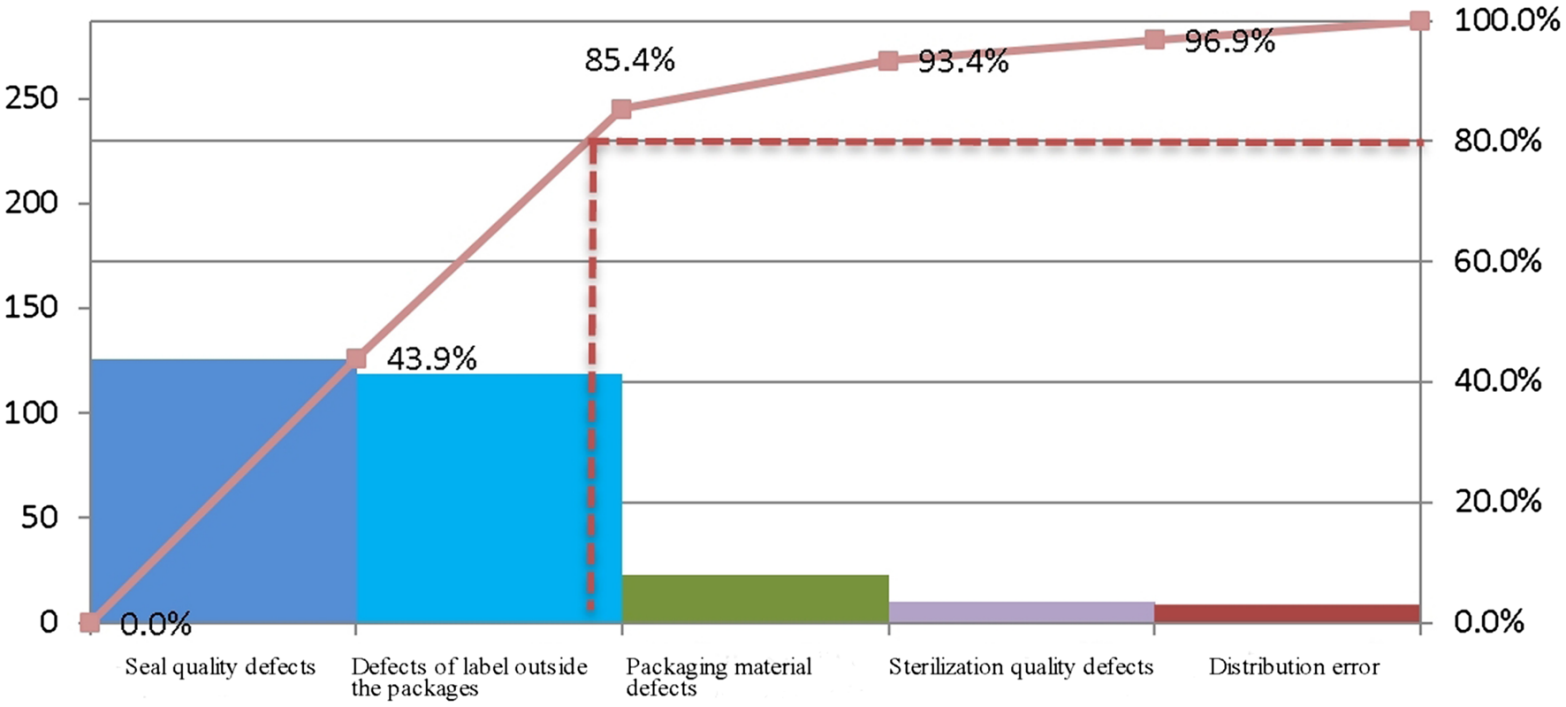
Classification of distribution defects among the sterile packages.

Pareto analysis was conducted on the data concerning the sterile packages distributed during May and June 2021 (Fig. 1). According to the 80/20 Rule, seal quality defects and defects of the label outside the package accounted for 43.9% and 41.5%, respectively, and were the main kinds of distribution defects found among sterile packages.

#### Understand (U)

According to the CQI team’s findings, the distribution defects among the sterile packages are characterized not only by defects in quality of the distribution work but also by defects in packaging and sterilization. Therefore, it is necessary to improve the quality of multiple processes in order to reduce the overall distribution defect rate. The CQI team analyzed the causes of seal quality and out-of-package label defects from five aspects: personnel, equipment, material, work procedure, and environment.

The main reasons of seal quality defects are as follows:

(1) *Personnel.* During plastic sealing, the packaging personnel did not always follow the standard plastic sealing workflow. In some cases, when the pouch was sent to the plastic sealing machine, the paper and the plastic layers of the pouch were not completely aligned and flattened, resulting in wrinkles and gaps in the sealing line, which potentially leads to sealing cracks^12^. Packaging personnel did not always carefully check the quality of sealing after finishing sealing. During sterilization loading, due to non-conforming loading, the plastic sealing pouch ended up being extruded and even damaged. Most of the distribution personnel were senior nurses, the average age of whom was over 45 years. Upon entering middle age, people’s eyesight generally starts to deteriorate, and it is likely that some personnel failed to assess visually the seal quality of the packages in some instances, and failed to identify sterile packages with non-conforming sealing within the fast-paced sterile package distribution framework.

(2) *Equipment.* In some cases the sealing temperature of the plastic sealing machine deviated beyond set margins. The system temperature of the plastic sealing machine has two modes: 125 °C, and 175 °C. If an incorrect temperature is selected for plastic sealing, the sealing quality will be non-conforming. If the sterilization trolley area is insufficient in size or the trolley surface is not smooth, the paper-plastic pouches will be scratched by the sterilization trolley during placement.

(3) *Material.* In some cases, the paper layer of the pouch was found to be thin and brittle, with insufficient tearing strength. If the pouch size is too small, or if the item is too large and heavy, or of irregular shape, or if multiple instruments are packaged in a paper-plastic pouch, the paper-plastic pouch is more likely to suffer damage.

(4) *Work procedure.* Errors in this category include imperfections in the distribution procedure, lack of detailed methods on paper-plastic package inspection, inappropriate plastic sealing implementation, and staff not being fully cognizant of the standard plastic sealing workflow.

(5) *Environment.* The CSSD is located on a low floor of the building, illuminated by old lamp tubes which provide poor lighting, resulting in lack of light in the distribution area. Under this condition, the distribution personnel might not always identify seal quality defects timeously during quality inspection.

The main reasons of defects of label outside the package are as follows:

(1) *Personnel.* Packaging personnel might not always carefully check whether the label information is consistent with the items inside the package when labeling, and so mislabeling might occur. In some cases the packaging personnel might not recognize the label information or detect the wrong label in time; the sterilization personnel might not always follow guidelines during sterilization loading, leading to friction occurring between the label and the inner wall of the sterilizer, causing the label to slide off or become illegible. There were insufficient distribution personnel during peak hours, so staff members might face greater pressure in their efforts to check the label information accurately. Further, due to the frequent rotation of the distribution personnel, they might not be familiar with the quality inspection criteria of certain sterile package labeling in the early stage of handover.

(2) *Equipment.* The information in the tracking system was incomplete. Some label information was not inputted into the system when it was created, resulting in information loss on the printed label. Label printer fault could occur, causing printed information to be blurred and the tracking barcodes to appear distorted.

(3) *Material.* The label printing paper was sensitive to humidity and heat. During high temperature sterilization, high temperature steam could penetrate the label, causing the font to fade and blur. The label size might be too small, resulting in incomplete label printing with excessive contents. Or the label adhesiveness might be weak, resulting in the label falling off during delivery. Another material-related reason was improper packaging materials: to meet the needs of different clinical departments, the same sterile items were packaged using various packaging materials, such as cotton wrapper, non-woven fabric wrapper, or paper-plastic pouch. These different materials may bear the same label name but they have different validity periods, and incorrect material selection can lead to labeling errors. For example, items packaged using cotton wrapper are considered sterile for 14 days, but the cotton-wrapper package is labeled ‘180 days’, which is the valid storage period of the non-woven wrapper package. If the mislabeled package is distributed to the clinical department, nosocomial infections may occur^13^.

(4) *Work procedure.* The label printing procedure was complicated, and it should be borne in mind that many of the packaging personnel in our hospital were low-tier workers with low educational attainment. Label printing errors are likely to occur among less experienced personnel who are not fully cognizant with the label printing procedure. Label pasting methods had not yet been harmonized, and so different staff members tended to paste labels at different positions on the packages, which could impair the inspection process.

(5) *Environment.* The CSSD is located on a low floor of the building, illuminated by old lamp tubes which provide poor lighting, resulting in lack of light in the distribution area. Under this condition, the distribution personnel might not always identify the labeling defects timeously during quality inspection.

**Figure 2:**
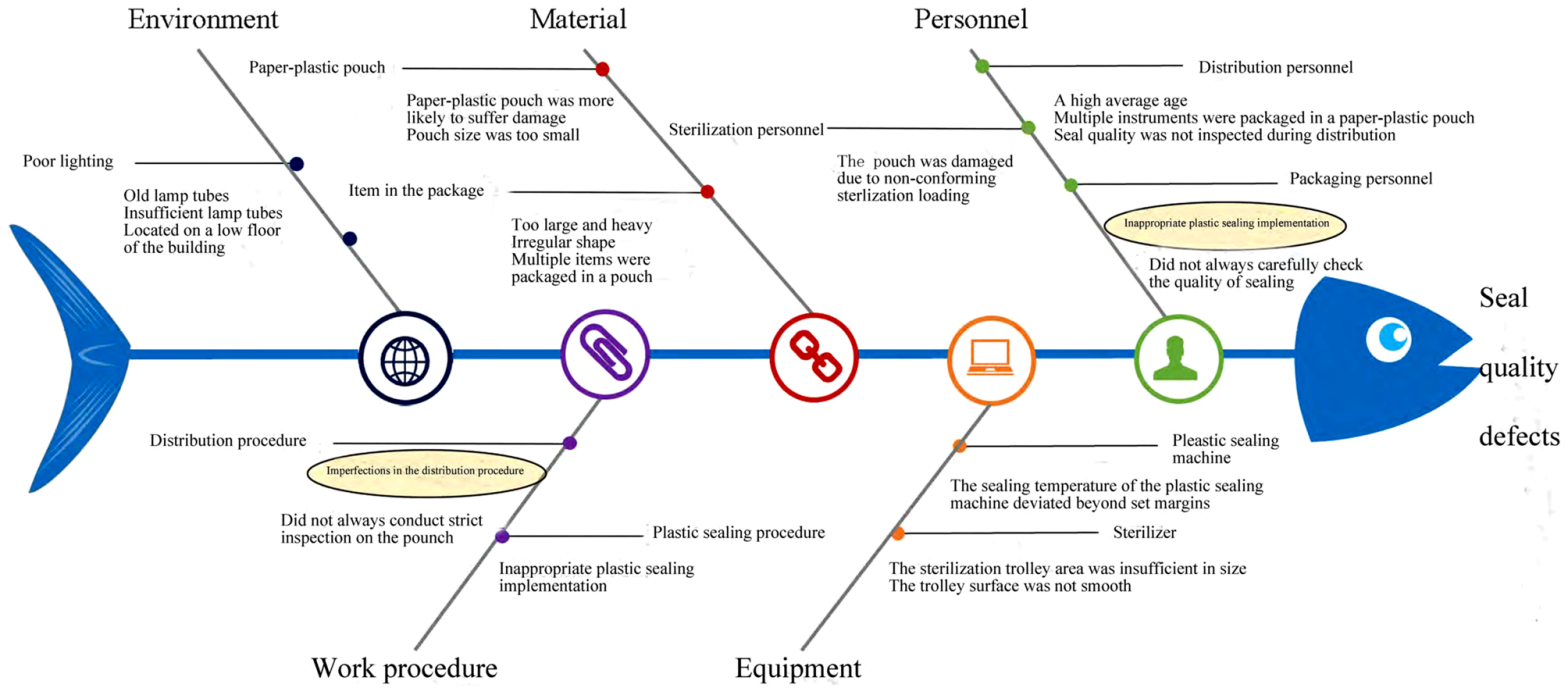
Root causes of seal quality defects.

**Figure 3:**
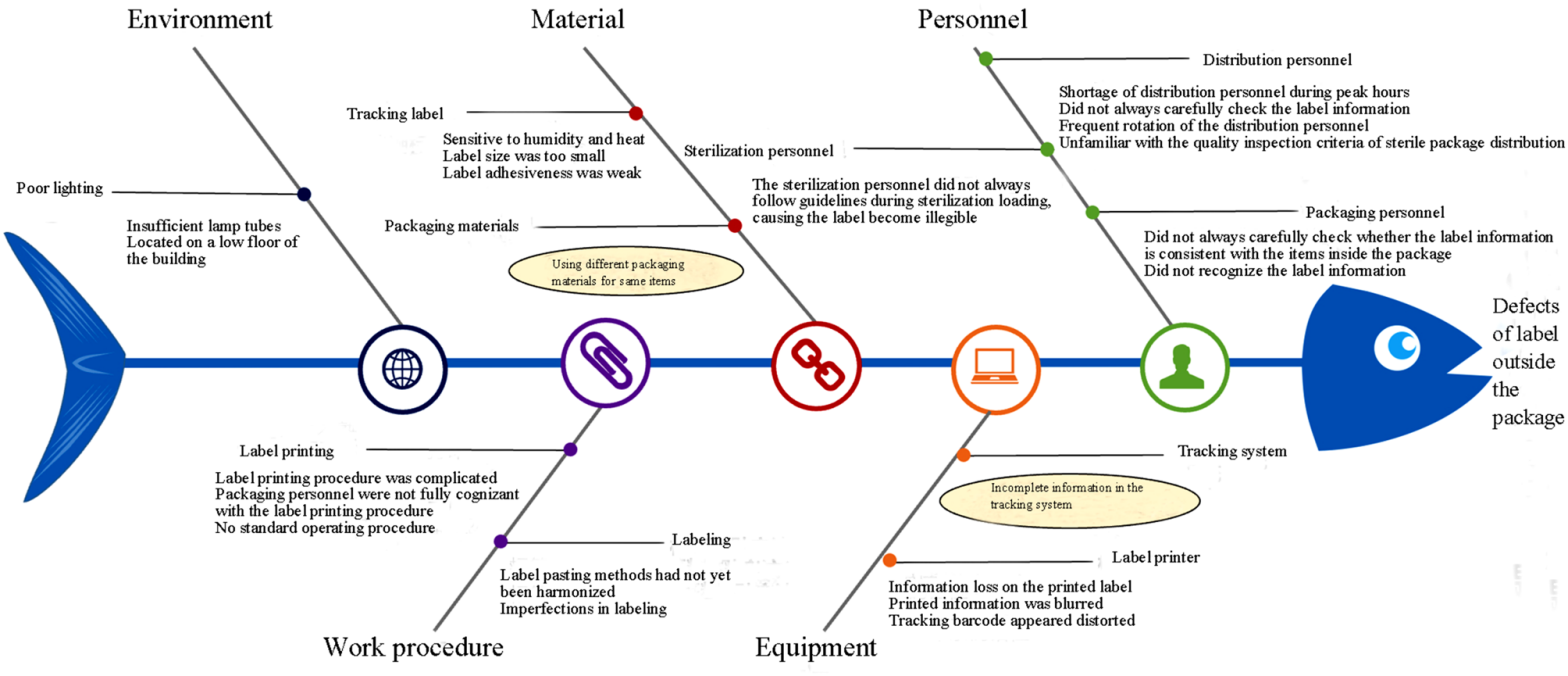
Root causes of defects of label outside the package.

The fishbone diagram was used to analyze the root causes of seal quality and labeling defects. As described above, 5 broad factors were identified (personnel, equipment, material, work procedure, and environment) during the verification of the true causes. Finally, 4 specific causes were determined: imperfections in the distribution procedure, inappropriate plastic sealing implementation, using different packaging materials for same items, and incomplete information in the tracking system (Figs. 2 and 3).

#### Select (S)

These 4 main reasons were identified by means of the fishbone diagram analysis. Upon these findings, the CQI team formulated the following improvement measures: (1) improve the distribution procedure; (2) develop quantitative criteria for plastic sealing, conduct work performance assessment, and ensure staff strictly abide by said plastic sealing criteria; (3) communicate with the clinical departments to arrive at a harmonization of packaging materials appropriate for different sterile items; and, (4) collaborate with the tracking system engineer to improve the label information in the tracking system.

#### Plan (P)

The CQI team pronounced the following formula for redressing the distribution defect rate: (1) refine the work procedure and implement identified solutions to reduce the distribution defect rate; (2) conduct training on workflow and relevant knowledge for relevant staff; (3) implement the improvement plan, and collect, sort, summarize and analyze the data regularly; (4) investigate the occurrence of distribute defects after intervention; and, (5) For aspects requiring improvement, formulate the next stage of the work plan and rectification measures.

#### Do (D)

**Figure 4:**
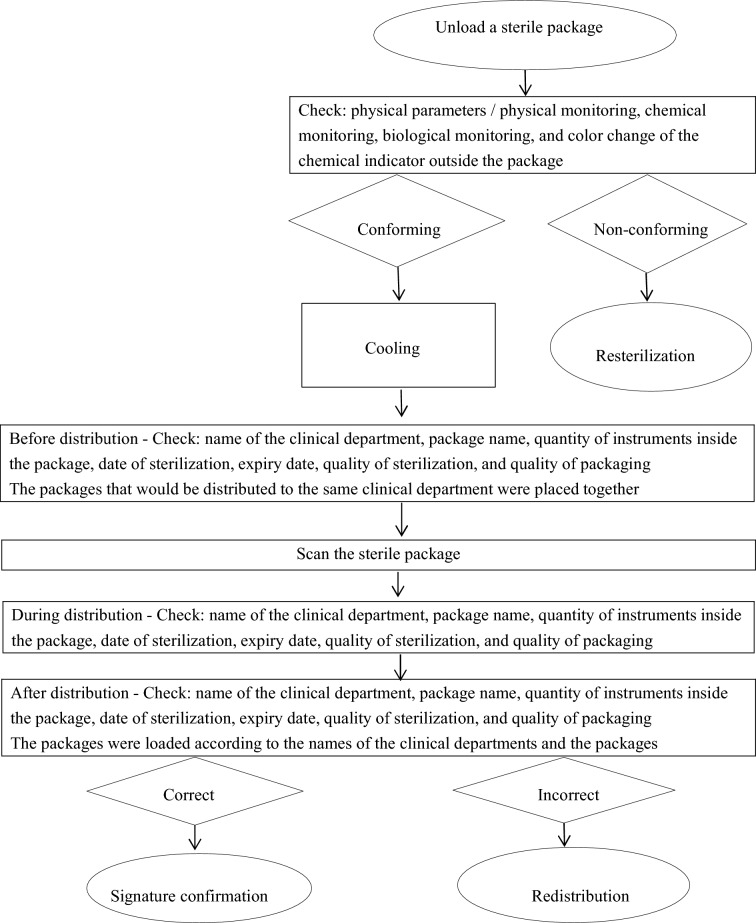
Distribution workflow.

The detailed implementation methods are as follows:

(1) *Revise the distribution management system and improve the distribution procedure* (Fig. 4). Following sterilization of items, distribution personnel must check all sterile items to ensure that the sterilization quality complies with relevant criteria prior to distribution to the clinical departments. Staff should determine accurately whether the color change of the chemical indicator outside the package complies with the criteria, whether the packaging material is clean, and whether the packaging tightness is appropriate^14^. If the sterilization quality is non-conforming, the package shall be re-sterilized^15^. During sterile package distribution, the ‘first-in, first-out’ principle should be followed. This is to prevent expiration of the package storage period—when the expiration date of the sterile package which first arrives at the sterile item storage area is earlier than that of the sterile package which arrives later^16^. The information (sterilization date, expiry date, name of clinical department, package name, and quantity of items inside the package, etc.) on the sterile package should be carefully checked prior to distribution^17^. Following these checks, the sterile packages shall be placed in the enclosed transport boxes or trolleys, and then distributed to the correct clinical departments. The head nurse should reasonably allocate human resources, appropriately increase the number of distribution personnel during peak hours, and seek to recruit young personnel with good eyesight and computer skills in a long-term strategy to improve distribution quality. New distribution recruits should possess excellent knowledge of aseptic concept as well as a good track record of relevant work experience.

(2) *Develop quantitative criteria for plastic sealing, conduct work performance assessment, and strictly implement the plastic sealing procedure.* Non-woven fabric wrapper, cotton wrapper, or other packaging methods where specified should be selected for items of an irregular shape, or which are oversized or overweight. Multiple instruments should be put in separate paper-plastic pouches. During plastic sealing, a paper-plastic pouch of appropriate size must be selected on the basis of the size of the sterile item^18^. There should be minimum 1 cm margin around the item inside the pouch, and the item inside the pouch should be 3–5 cm away from the pouch seal^19^. When sealing, one terminal of the pouch shall be sealed before the item is placed inside. Air inside the punch shall be eliminated prior to the paper and plastic layers of the other terminal being aligned and flattened into the conveyor belt. After sealing, the seal shall be checked to ensure that no wrinkles or bubbles occur. The head nurse and quality controllers shall randomly check the implementation of the packaging personnel to ensure that they implement plastic sealing in conformity with workflow.

(3) *Same packaging material for same items. *The packaging of different sterile items should accord with a harmonized system. Cotton wrappers should be replaced by non-woven wrappers to make sterile items remain sterile for 180 days, so as to prevent expiry of the storage period.

(4) *Improve the tracking system.* The current tracking label shall be replaced with a high temperature-resistant, waterproof label in an effort to prevent label illegibility following sterilization. All details of the sterile items shall be added to the tracking system in order to more accurately detect instances of incomplete label information.

(5) *Complete the training for all staff to improve their working competence*. Specific personnel are designated to be responsible for training and assessment. The training is carried out combining theoretical knowledge with scene simulation. The assessment is conducted by inspecting the distribution process and random inspection on the distributed items.

(6) *Create a quality control register.* A quality control register shall be created to accurately record problems occurring in the CSSD workplace in order to ensure timely correction of problems arising as well as detection of hidden problems.

#### Check (C)

The following checking procedures have been formulated:

1. Clarify the responsible person, and continue to track to ensure appropriate implementation.
2. CQI team members should strengthen checking procedures to ensure that packaging personnel appropriately implement and adhere with the packaging and plastic sealing procedure, and to ensure that the distribution personnel strictly check the distribution. A distribution defect check sheet shall be created to record the categories and causes of the defects.
3. Personnel will be tasked with periodically analyzing data to determine the causes of distribution defects and taking measures for continuous quality improvement.

#### Act (A)

To solve any problems, the next cycle of continuous quality improvement may be conducted.

### Observation indicators

Following implementation of the FOCUS-PDCA quality improvement model for distribution defect intervention, the classification and rates of distribution defects and clinical departments’ satisfaction level to CSSD work were compared.

### Statistical methods

SPSS20.0 was used for data analysis. The enumeration data are presented as ratios. The Chi-square test was used for drawing comparisons among defect rates and defect categories. The *P* < 0.05 indicates a statistically significant difference. The hypothesis testing on defect rates and defect categories is as follows: the two groups of defect rates/defect categories (incidence rates of sealing quality defects, defects of label outside the package, packaging material defects, sterilization quality defects, and distribution error) were the same; degree of freedom = 1. The statistical test equation is as follows (A refers to actual frequency and T refers to theoretical frequency):

$$\chi^{2} = \sum {\frac{{(A - T)^{2} }}{T}}$$

The measurement data are presented as (mean ± standard deviation). The independent two-sample *t*-test was applied to identify differences among the clinical departments’ satisfaction scores on CSSD work before and after the application of the FOCUS-PDCA quality improvement model (a statistically significant difference exists when *P* < 0.05). The hypothesis testing on the satisfaction scores is as follows: the satisfaction scores of the two groups were the same; degree of freedom = 31. The statistical test equation is as follows ($\overline{X}1$ and $\overline{X}2$ refer to means of the two samples; $S_{C}^{2}$ refers to pooled variance):

$$t = \frac{{\overline{X}_{1} - \overline{X}_{2} }}{{\sqrt {S_{C}^{2} \left( {\frac{1}{{n_{1} }} + \frac{1}{{n_{2} }}} \right)} }}\quad v = n_{1} + n_{2} - 2$$

### Ethics approval and consent to participate

This study was conducted in accordance with the Declaration of Helsinki. All research methods were carried out in accordance with the relevant guidelines and regulations. This study was approved by the Medical Ethics Committee of West China Second University Hospital, Sichuan University [2023 Medical Scientific Research for Ethical Approval No. (001)]. Verbal informed consent to participate in this study was obtained from all participants. The Medical Ethics Committee of West China Second University Hospital, Sichuan University approved the procedure of verbal informed consent of this study.

## Results

Data for distribution defect rates among the control and observation groups are presented in Table 1. The hypothesis testing on distribution defect rates was conducted to see if the two groups of defect rates were the same. Statistical reference was used for the Chi-square test of four-fold table in a completely randomized design. A statistically significant difference was identified by χ^2^ = 144.82 and *P* < 0.001. It can be considered that a difference in distribution defect rates existed between the two groups. The distribution defect rate of sterile packages in the observation group was 0.37%, lower than that in the control group (1.74%).

**Table 1:** Distribution defect rates among the control and observation groups.

Groups	Number of the distributed sterile packages	Number of the sterile packages with distribution defects	Distribution defect rate %
Control group	16,480	287	1.74
Observation group	15,976	59	0.37

Remarks: χ^2^ = 141.8, *P* < 0.001.

Classification of distribution defects of the sterile packages of the control and observation groups are presented in Table 2. The hypothesis testing on defect categories was conducted to see if the two groups of defect categories (sealing quality defects, defects of label outside the package, packaging material defects, sterilization quality defects, and distribution error) were the same. Statistical reference was used for the Chi-square test of four-fold table in a completely randomized design. For sealing quality defects, a statistically significant difference was identified by χ^2^ = 54.85 and *P* < 0.001. It can be considered that a difference in incidence rates of sealing quality defects existed between the two groups. The incidence rate of sealing quality defects in the observation group was 0.19%, lower than that in the control group (0.76%). For defects of label outside the package, a statistically significant difference was identified by χ^2^ = 71.68 and *P* < 0.001. It can be considered that a difference in incidence rates of defects of label outside the package existed between the two groups. The incidence rate of defects of label outside the package in the observation group was 0.11%, lower than that in the control group (0.72%). For packaging material defects, a statistically significant difference was identified by χ^2^ = 8.05 and *P* < 0.05. It can be considered that a difference in incidence rates of packaging material defects existed between the two groups. The incidence rate of packaging material defects in the observation group was 0.04%, lower than that in the control group (0.14%). For sterilization quality defects, a statistically significant difference was identified by χ^2^ = 3.87 and *P* < 0.05. It can be considered that a difference in incidence rates of sterilization quality defects existed between the two groups. The incidence rate of sterilization quality defects in the observation group was 0.01%, lower than that in the control group (0.06%). For distribution error, a statistically significant difference was identified by χ^2^ = 4.69 and *P* < 0.05. It can be considered that a difference in incidence rates of distribution error existed between the two groups. The incidence rate of distribution error in the observation group was 0.01%, lower than that in the control group (0.05%).

**Table 2:** Classification of distribution defects of the sterile packages.

Groups	Number of the sterile packages	Classification of distribution defects				
		Seal quality defects	Defects of label outside the package	Packaging material defects	Sterilization quality defects	Distribution error
Control group	16,480	126	119	23	10	9
Observation group	15,976	31	18	7	2	1
χ^2^		54.85	71.68	8.05	3.87	4.69
*P*		< 0.001	< 0.001	< 0.05	< 0.05	0.05

Data detailing clinical departments’ satisfaction levels before and after application of the FOCUS-PDCA quality improvement model are presented in Table 3. The hypothesis testing on satisfaction levels was conducted to see if the clinical departments’ satisfaction scores on CSSD work before and after application of the FOCUS-PDCA quality improvement model were the same. Statistical reference was used for the two-sample *t*-test in a completely randomized design. A statistically significant difference was identified by t = 6.94 and *P* < 0.001. The clinical departments’ satisfaction score on CSSD work after the application of the FOCUS-PDCA quality improvement model was (98.07 ± 0.28), higher than that before the application of the FOCUS-PDCA quality improvement model (95.53 ± 0.24).

**Table 3:** Clinical departments’ satisfaction levels to CSSD work before and after application of the FOCUS-PDCA quality improvement model.

Indicator	Score before application of the FOCUS-PDCA quality improvement model	Score after application of the FOCUS-PDCA quality improvement model	t	*P*
Satisfaction levels	95.53 ± 0.24	98.07 ± 0.28	6.94	< 0.001

## Discussion

### The reduction in distribution defect rate of sterile packages is conducive to improving the quality of medical care

The quality of sterile packages and efficiency of package distribution provided by the CSSD are important factors affecting the safety of clinical medical work^20^. Our hospital’s CSSD provides approximately 2000 sterile packages for 32 clinical departments every day. Due to the imperfections in the distribution procedure, inappropriate implementation of plastic sealing, and same instruments being placed inside different packaging materials, the distribution defect rate was found to be as high as 1.74%. Distribution defects of sterile packages lead to prolonged waiting time for patients, medical accidents and even nosocomial infections^21^. It is necessary for the CSSD to identify and isolate non-conforming sterile packages for re-processing^22^, a target which requires additional manpower and financial investment, which in turn will lead to improvements in overall work efficiency and clinical departments’ satisfaction with CSSD work. The CSSD staff are required to strictly implement and adhere with national criteria and further improve the quality of their work to eliminate distribution defects.

### The FOCUS-PDCA quality improvement model is an effective approach to improve the quality of sterile package distribution

The PDCA quality improvement model, which is divided into 4 steps (Plan, Do, Check, and Act), has been widely used in healthcare field in China and abroad^23^. The FOCUS-PDCA quality improvement model adds 5 steps on the basis of PDCA, namely Find (F) a problem to improve, Organize (O), Clarify (C), Understand (U), and Select (S). The model can analyze each step of the work process in more detail to achieve the purpose of continuous improvement^24^.

As a quality management tool, the FOCUS-PDCA quality improvement model has played a significant role in hospital management system building, clinical nursing and ward management^25, 26^. Sun et al.^27^ reported that the FOCUS-PDCA program could reduce the incidence of wet packs of foreign medical devices from 4.32 to 0.72%. In our study, the FOCUS-PDCA quality improvement model was applied to the continuous improvement of the quality of sterile package distribution. Problems were identified according to the FOCUS model, the current situation was improved and a CQI team was established to analyze the causes of the distribution defects, combined with the fishbone diagram. Then, the solutions were developed and implemented using PDCA. The results of our study showed that the distribution defect rate of sterile packages was reduced from 1.74 to 0.37% after using the FOCUS-PDCA quality improvement model.

### The application of the FOCUS-PDCA quality improvement model can make the management and quality control of sterile package distribution more scientific

The FOCUS-PDCA quality improvement model is a scientific quality improvement method and an active behavior to seek quality improvement^28^. A professional team is set up according to the problems and the team members discuss and analyze the problems. The team consults relevant criteria, guidelines, expert consensus and literature^29^ to further improve the quality criteria of sterile package distribution. The team review whether there are defects in the sterile package distribution process, then revise and solidify the defective work steps, thereby making the sterile package distribution more scientific and standardized.

### The application of FOCUS-PDCA quality improvement model can improve CSSD’s image

If sterile packages with quality defects are distributed to clinical departments, the CSSD’s image could be damaged if the departments think that CSSD staff lack professional knowledge, skills and responsibility. The incidences of distribution defects in sterile packages were significantly reduced after the application of the FOCUS-PDCA quality improvement model, and the sterile packages with quality defects were processed within the CSSD, thereby avoiding a bad impression. The results of our study have shown that the clinical departments’ satisfaction level on CSSD work was significantly improved after the application of the FOCUS-PDCA quality improvement model, thus improving CSSD’s image.

### Limitations

Our study applied the FOCUS-PDCA model to improve the quality of sterile package distribution. The survey data were only from our hospital, so they could not represent the distribution defects in other hospitals. Moreover, our study only analyzed the first two causes of distribution defects. The causes of packaging material defects, sterilization quality defects and distribution error, also need to be studied. Therefore, our study has limitations in improvement measures for distribution defects. In future research, it is necessary to improve the breadth of data collection and make a comprehensive analysis on the quality of CSSD work to propose more comprehensive improvement measures.

## Conclusions

The FOCUS-PDCA quality improvement model is a circular improvement process which can effectively reduce the incidence of sterile package distribution defects, ensure the provision of high-quality sterile packages and patient safety, and reduce the risk of nosocomial infections. It can also improve the team’s competence in identifying and solving problems and help to develop their scientific thinking in solving problems that arise.

## Data Availability

The datasets used and/or analysed during the current study are available from the corresponding author on reasonable request.

## Abbreviations

CSSD: Central sterile supply department
CQI: Continuous quality improvement
